# Aneurism of Aorta, and Rupture within the Pericardium

**Published:** 1849-02

**Authors:** C. L. Ford

**Affiliations:** Buffalo


### *Aneurism of Aorta, and Rupture within the Pericardium.*

Dr. Flint,

*Dear Sir.*—The following sketch of pathological appearances observed in a subject at the anatomical rooms of the Medical College during the current course, is at your disposal.

The subject was a female, aged, perhaps, 50, rather fleshy, presenting nothing especially worthy of note except the following:—

On removing the sternum and costal cartilages, the pericardium was observed to be very much distended. Section through it exposed a pint or more of coagulated blood, occupying a position in front, and at the right side of the heart.

On examining for its source, I found a rupture of the ascending aorta two inches from its origin. The rent was perpendicularly in the artery, nearly straight, and three-fourths of an inch in length.

Proceeding upwards, I found a very large aneurism, principally of the ascending aorta, though the arch, and descending aorta, were much above their normal size.

The sack was nearly filled with coagulated blood. On removing this, and moderately distending the parts, I made the following measurement;—

| | |
|---|---|
| From the commencement of the aorta, around the greatest prominence of the sack, to the origin of the arteria innominata, | 7½ inches. |
| Circumference of the sack, | 9½ " |
| Size of aorta close to a innominata, | 5 " |
| Size of aorta beyond left subclavian, | 5½ " |
| Lower portion of thoracic aorta, | 3¾ " |
| At origin of cœliac artery, | 3½ " |
| At bifurcation into iliacs, | 2½ " |
| Length of arteria innominata, | 2 " |
| Circumference at origin, | 3¾ " |
| Circumference an inch above, | 3½ " |
| Origin of pulmonary artery, | 4 " |

The dilatation was mostly on the right side, and front, of the ascending aorta. The surface was rough, apparently covered with fibrinous deposit. The descending vena cava adhered intimately to it. The pericardium adhered to the upper part of the sack, but the lower portion, where the rupture occurred, and some distance above it, had no such adventitious aid.

The thickness of the walls varied little from the normal state, though in the immediate vicinity of the laceration, the coats were obviously thinner.

The heart, though large, did not appear to be particularly diseased. Such is a brief sketch of the appearances presented in this interesting case, from which, it will be observed, that the ascending aorta was most largely dilated; that the arch, and upper portion of the thoracic aorta were also greatly enlarged; that almost the entire length of the aorta was implicated in the disease, which, in descending, became gradually less prominent. The arteria innominata was likewise much dilated, but the arteries derived from it appeared not above the normal size. The other vessels from the arch presented nothing unusual.

The preparation may be seen in the Museum of the Medical Department of the University of Buffalo.

Respectfully, yours,

Buffalo, Jan. 15, 1849. C. L. FORD.

